# Trajectories of Loneliness During Adolescence Predict Subsequent Symptoms of Depression and Positive Wellbeing

**DOI:** 10.1007/s10964-023-01925-0

**Published:** 2023-12-21

**Authors:** Simon C. Hunter, Rebecca Seth, Stephen Houghton, David Lawrence, Corinne Zadow, Michael Rosenberg, Lisa Wood, Pamela Qualter, Trevor Shilton

**Affiliations:** 1 Glasgow Caledonian University, Cowcaddens Road, Glasgow, G4 0BA Scotland UK; 2 University of Western Australia, 35 Stirling Highway, Crawley, 6009 WA Australia; 3 Curtin University, Kent Street, Perth, WA 6102 Australia; 4 University of Strathclyde, 16 Richmond Street, Glasgow, G1 1XQ Scotland UK; 5 University of Notre Dame, 23 High Street, Fremantle, WA Australia; 6 University of Manchester, Oxford Road, Manchester, M13 9PL England UK

**Keywords:** Loneliness, Trajectories, Depression, Positive Mental Wellbeing

## Abstract

There is a need to identify the outcomes of changes in loneliness during adolescence, and to consider this within a multidimensional framework of loneliness. This study considered the effects of different trajectories of change in Isolation Loneliness and in Friendship Loneliness upon both positive wellbeing and symptoms of depression. To achieve this, 1782 (43% female; 12.92 years old at the start of the study, SD = 1.60) young people took part in a longitudinal study with four data points across 2 years. Four Isolation Loneliness trajectories and five Friendship Loneliness trajectories were identified. Youth who experienced low levels of Isolation Loneliness that subsequently increased appear to be at particular risk for poor outcomes. Similarly, initially high levels of Friendship Loneliness that decreased rapidly, or which began at a low level and only increased marginally, seem to also be a risk. Loneliness is a multi-dimensional construct and its development during adolescence impacts upon young people’s depressive symptomatology and positive mental wellbeing.

## Introduction

Loneliness is associated with numerous indices of maladaptive adjustment and there is an attendant need to identify the outcomes of changes in loneliness during adolescence. Empirical study has begun to investigate these relationships but has neither considered positive mental wellbeing as an outcome nor addressed the multidimensional nature of loneliness. This study identified trajectories of change in relation to two dimensions of loneliness (Isolation loneliness and Friendship loneliness) and considered the effects of these on both positive mental wellbeing and symptoms of depression.

Loneliness is the sense that one’s social relationships are not commensurate with one’s desired social relationships (Cacioppo et al., 2015a). Considering loneliness across the life-span, it is evident that adolescence and early adulthood are times when loneliness peaks (Heinrich & Gullone, 2006; Xerxa et al., 2023). According to the Office for National Statistics (2018) nearly half of 10–12-year-olds report feeling lonely at least some of the time, with this rising to almost 60% in 16–24-year-olds. Brain development during adolescence in regions involved in social processing, a desire for increased peer interaction and friendships (Orben et al., 2020), but increased sensitivity to social rejection (Blakemore & Mills, 2014), and developmental shifts and transitions in social networks (e.g., moving from primary to secondary school, or leaving home) are all known risk factors for increased loneliness (Siva, 2020; Sundqvist & Hemberg, 2021).

Adolescence is a particularly vulnerable period for the development of mental health problems, with half of all mental health problems emerging before the age of 14 (Kessler et al., 2007) and the peak age of incidence coinciding with the transition from “childhood/adolescence” to “adult” life (Thapar & Riglin, 2020). Loneliness in adolescence is a known risk factor for anxiety (Maes et al., 2019), depression (Fontaine et al., 2009; Lasgaard et al., 2011), suicidal ideation (Gallagher et al., 2014) and diminished positive mental health (Lyyra et al., 2021). Moreover, loneliness is associated with later reports of mental health problems, physical health risk behaviours, poorer employment prospects (Leigh-Hunt et al., 2017; Matthews et al., 2018), poorer sleep quality (Matthews et al., 2017), withdrawal from social activities and relationships (Böger & Huxhold, 2018), suicidal behaviours (Heinrich & Gullone, 2006), cardiovascular disease (Leigh-Hunt et al., 2017) and both morbidity and mortality (see Hawkley & Cacioppo, 2010). Given that loneliness experienced in adolescence can have both immediate and lasting implications for outcomes in later years, studying loneliness in young people is important.

The degree to which different trajectories of loneliness are associated with psycho-social adjustment has often been the focus of published work (see Table 1). Confidently naming trajectories relating to loneliness is problematic since there is no accepted definition of low, medium, or high loneliness. However, across studies, youth in stable-low trajectory groups tend to report better psychological and social adjustment than those in other groups, including lower depressive symptomatology (Jobe-Shields et al., 2011; Ladd & Ettekal, 2013; Qualter et al., 2013; Schinka et al., 2013; Vanhalst et al., 2013), higher social skills (Schinka et al., 2013), better self-esteem (Vanhalst et al., 2013), and higher academic outcomes (Benner, 2011). Young people with chronically high levels of loneliness tend to be most at risk for depressive symptomatology (Ladd & Ettekal, 2013; Qualter et al., 2013; Schinka et al., 2013; Vanhalst et al., 2013), alcohol misuse (Qualter et al., 2013), suicidal ideation (Schinka et al., 2013), and poorer general health (Harris et al., 2013; Qualter et al., 2013). Among those with trajectories of loneliness that display change over time, there is evidence that increasing levels of loneliness are a marker for later maladjustment (Benner, 2011; Jobe-Shields et al., 2011; Qualter et al., 2013) and there is some support for a scar hypothesis (Rohde et al., 1990) whereby higher starting levels that subsequently reduce are still associated with poor outcomes (Harris et al., 2013).

**Table 1:** Studies estimating trajectory classes of loneliness among children and young people

Study	Sample	Design	Trajectory classes^a^	Results
Harris et al. (2013)	*N* = 209. Aged 8 years at T1.	Data collected three times, at 18-month intervals.	Two-class solution: Relatively High, Reducing (48%), and Low Stable (52%).	At 11 years, the Relatively High, Reducing group had higher levels of depressive symptoms, poorer general health, and poorer sleep.
Benner (2011)	*N* = 640. Grade 9 at T1.	Data collected twice in Grades 9 and 10.	Three-class solution: Consistently Low (78%), High (11%), and Low but Increasing (11%).	High and Low Increasing classes had poorer academic outcomes than the Low class.
Jobe-Shields et al. (2011)	*N* = 170. Aged 9 years at T1.	Data collected annually at ages 9, 10 and 11 years.	Three-class solution: Stable Low (65%), Increasers (23%), and Decreasers (12%).	Stable Low had generally positive peer functioning; Increasers at risk group for developing later internalising symptomatology; and Decreasers had mixed pattern of peer functioning at age 9 years, but were indistinguishable from the Stable Low group subsequently.
Verboon et al. (2022)	N-130. 9 years.	Data collected at ages 9, 13, 16, and 21.	Three-class solution: Stable-Low (51–61%), Low-Increasing (21–32%), and High-Declining (7–22%). *NB. Ranges are reported here as three separate clustering techniques were employed*.	
Qualter et al. (2013)	*N* = 586. Age 7 years at T1.	Data collected biannually for ten years.	Four-class solution: Low Stable (37%), Moderate Decliners (23%), Moderate Increasers (18%), Relatively High Stable (22%).	Both the High Stable and Moderate Increasing trajectories were associated with depressive symptoms at age 17 years.
Ladd & Ettekal (2013)	*N* = 478. Age 12 years at T1.	Data collected annually for six years.	Five-class solution: Stable Non-Lonely (19%), Stable Low (20%), Stable High (Chronic) (14%), Moderate Decliners (42%), and Steep Decliners (6%).	Stable High had the highest level of symptoms of depression.
Schinka et al. (2013)	*N* = 832. Age 9 years at T1.	Loneliness assessed at ages 9, 11, and 15 years.	Five-class solution: Stable Low (49%), Moderate Increasing (32%), High Increasing (5%), Decreasing (11%), and Chronic (4%).	Depression assessed at 7 years old predicted greater likelihood of being in: High Increasing/Chronic groups than Stable Low. All groups reported higher levels of depression at age 15 when compared to the Stable.
Vanhalst et al. (2013)	*N* = 389. M_age_ at T1 = 15.22 years.	Cohort-sequential (two-groups, ages 15 and 16 at T1). Data collected annually for 5 years.	Five-class solution: Chronically High (3%), High Decreasing (6%), Moderate Decreasing (8%), Low Increasing (17%), and Stable Low (65%).	Those who never experienced loneliness were best adjusted (lowest stress, least depressive symptoms, highest self-esteem), whereas the Chronically High group had the most problems (highest stress, most depressive symptoms, lowest self-esteem).
Vanhalst et al. (2015, 2018)	*N* = 730. M_age_ at T1 = 15.43 years.	Data collected annually for 4 years.	Five-class solution: Chronic (3%), Low Stable (47%), Moderate-Stable (27%), Moderate-Increasing (14%), High Decreasing (9%).	Cognitive and emotional responses of the Chronic group seem to perpetuate, rather than reduce, loneliness (e.g., hypersensitivity to social exclusion and hyposensitivity to social inclusion).

^a^Trajectory labels used here are those used by the original authors

As is clear from Table 1, symptoms of depression have frequently been the focus of research seeking to document the outcomes of different loneliness trajectories. However, notably absent from this literature examining trajectories of change in loneliness is information concerning associations with positive mental wellbeing. Positive mental wellbeing is neither the polar opposite, nor absence, of psychological maladjustment (World Health Organisation WHO (2004)) and includes both hedonic (i.e., happiness, subjective well-being) and eudemonic (i.e., positive functioning) aspects of wellbeing (Clarke et al., 2011). Promoting positive mental wellbeing among adolescents is a national priority in many countries and has been placed at the centre of Government policy involving children and young people in the UK (Garrat et al., 2022; Scottish Government, 2020). Cross-sectional work with adolescents has supported that a negative association exists between positive mental wellbeing and loneliness (Houghton et al., 2016; Lyyra et al., 2021). Cross-lagged panel analyses using longitudinal data collected across the COVID-19 pandemic, in contrast, indicates that levels of loneliness may not be associated with positive mental wellbeing (Houghton et al., 2022).

While positive mental wellbeing is negatively correlated with indices of psychological maladjustment such as depressive symptomatology (Clarke et al., 2011) it is also the case that depressive symptomatology is more strongly associated with subsequent positive mental wellbeing than vice-versa among adolescents (Zadow et al., 2017). Indeed, in adolescence, low levels of positive mental wellbeing can occur in the presence of low levels of depressive symptomatology and high levels of positive mental wellbeing can occur even in the presence of mental illness (Patalay & Fitzsimons, 2018), and there is significant change between groups over time (Petersen et al., 2022). Thus, negative and positive mental wellbeing are distinctive concepts and the degree to which tackling loneliness can contribute to improving either health outcome is not clear.

As well as focussing only on negative indices of adjustment as outcomes of loneliness, the current literature examining trajectories of loneliness has not addressed the multi-dimensional nature of the construct. Contemporary theory and measurement of loneliness considers it to have between two and four dimensions (Goossens et al., 2009; Houghton et al., 2014; Majorano et al., 2015). Parent (or family)-related and peer-related loneliness sub-scales have been reported in two separate studies (Goossens et al., 2009; Marjorano et al., 2015), and to these have been added two attitudinal factors reflecting positive and negative attitudes toward solitude (Goossens et al., 2009). While there exists support for the existence of positive and negative attitudinal factors, they may reflect attitudes toward “aloneness” (e.g., “I have discovered the benefits of being alone”) rather than loneliness per se (Houghton et al., 2014). Furthermore, parent (or family)-related and peer-related factors risk confusing the construct with the situations in which it may be expressed and thus it has been proposed that factors relating to friendship loneliness (e.g., “I feel part of a group of friends”, where higher scores reflect a positive outcome) and isolation loneliness (e.g., “I have nobody to talk to”, where higher scores reflect a negative outcome) are more theoretically distinct constructs (Houghton et al., 2014). There is clear evidence that feelings of isolation and quality of friendships are highly, inversely correlated among adolescents (Houghton et al., 2020).

Studying both isolation and friendships during adolescence is important because it is a period where belonging to a peer group is a major concern among young people (Rubin et al., 2008) and peer interactions and relationships become increasingly more important (Qualter et al., 2015; Rubin et al., 2009). For some young people, insufficient connections to others can lead to profound and long-standing negative consequences, while having quality friendships can provide numerous social and emotional benefits (Houghton et al., 2014). The turbulence of significant life transitions during adolescence means some young people drift in or out of loneliness while others experience loneliness persistently (Matthews et al., 2023). Cross-sectional research with adolescents shows that positive mental wellbeing is positively associated with “friendship” loneliness (where higher scores represent lower loneliness) and is negatively associated with “isolation” loneliness (where higher scores represent higher loneliness) (Houghton et al., 2016). This speaks to the importance of considering loneliness as a multi-dimensional construct, which no published work on loneliness trajectories and adjustment has yet considered.

## Present Study

There is a need to identify the outcomes of changes in loneliness during adolescence, and to consider this within a multidimensional framework of loneliness. The present study considers the effects of different trajectories of change in two forms of loneliness (isolation loneliness and friendship loneliness) upon both positive wellbeing and symptoms of depression. Based on existing research utilising single-factor loneliness scales, it is expected that four or five trajectories of loneliness will be evidenced for both isolation loneliness and friendship loneliness. The largest trajectory group in both cases is expected to be a consistently unproblematic loneliness trajectory (i.e., low isolation loneliness, high friendship related loneliness). A chronically problematic trajectory in each case (i.e., high isolation loneliness, low friendship related loneliness) is also expected. Based on the two existing studies that most closely resemble the present study in terms of participant age, it is expected that there will be a third trajectory reflecting loneliness which is problematic but subsequently becomes less so (isolation loneliness decreasing, friendship loneliness increasing). After controlling for earlier levels of both positive mental wellbeing and symptoms of depression, it is expected that youth in the chronically problematic loneliness trajectory groups will report significantly higher symptoms of depression, and significantly lower levels of positive mental wellbeing, than youth in the other trajectory groups.

## Methods

### Participants

At T1 (see Procedure for details of data collection points), there were 1544 youth (45% female), at T2 there were 1703 (45% female), at T3 there were 1782 (43% female), and at T4 there were 1591 (44% female). Average age at T1 was 12.92 years old (SD = 1.60; range = 10 to 16) and across the four waves of data collection 77–80% of young people reported living in an urban environment. Numbers of participants varied somewhat across waves because young people were invited to participate if they joined classes that were already involved in the study. One thousand two hundred and twelve participants took part in all four waves, while 524 took part in three, 101 took part in two, and 83 took part in a single session

Participants attended 38 separate schools (*N* = 34 State Government; *N* = 4 non-Government) which themselves reflected a range of socio-economic status (SES) areas as indexed by their Socio-Economic Index for Areas (Australian Bureau of Statistics, 2011). All eligible students were invited to participate and both information sheets and consent forms were sent to parents of students explaining that involvement would consist of data gathering over approximately three school years. Five primary schools were in low SES areas, three in mid SES areas, and five were in high SES areas. Among the high schools, three in low SES areas, nine in mid SES areas and 13 in high SES areas.

### Measures

#### Loneliness

The self-report Perth A-Loneliness scale (PALs: Houghton et al., 2014) which includes both a friendship related loneliness (e.g., “*My friends will stand by me in almost any difficulty*”, “*I feel part of a group of friends*”), and isolation loneliness (e.g., “*I feel like I do not have a friend in the world*”, “*I am not close to anyone*”) scale was utilised. Both scales comprised six items and responses are recorded using a six-point Likert scale (1 = “*never*”, 2 *=* “*rarely*”, 3 *=* “*sometimes*”, 4 *=* “*often*”, 5 *=* “*very often*”, 6 *=* “*always*”). Factor scores were obtained by summing the relevant items so that scores could range from 6 to 36, and Cronbach’s alpha was good for both scales (α_T1friendship_ = 0.91, α_T2friendship_ = 0.91; α_T3friendship_ = 0.92; α_T4friendship_ = 0.92; α_T1isolation_ = 0.87, α_T2isolation_ = 0.91, α_T3isolation_ = 0.91, α_T4isolation_ = 0.92). Higher scores indicated higher levels of the relevant construct (i.e. “worse” loneliness with regards to Isolation loneliness but “better” loneliness with respect to Friendship loneliness).

#### Symptoms of depression

The Children’s Depression Inventory 2 *(CDI 2*: Kovacs, 2004) is a 12-item self-report assessment of cognitive, affective, and behavioural symptoms of depression in children and adolescents aged 7–17 years. Each item has three separate sentence response options which describe participants’ feelings and experiences over the past 2 weeks (e.g., 0 = “*I am sad once in a while*”, 1 = “*I am sad many times*”, or 2 = “*I am sad all the time*”). One item on this scale was not used when calculating scale scores for participants since it related to feelings of loneliness: “*I do not feel alone, I feel alone many times, I feel alone all the time*”. Scores could range from 0 to 22, with higher scores reflecting higher depressive symptomatology, and the scale evidenced good internal reliability (α_T1_ = 0.81, α_T4_ = 0.85).

#### Positive mental wellbeing

The Short Warwick-Edinburgh Mental Wellbeing Scale (SWEMWBS: Stewart-Brown et al., 2009) is a 7-item self-report scale related to positive feelings, answered on a five-point Likert scale (1 = “*none of the time*”, 2 = “*rarely*”, 3 = “*some of the time*”, 4 = “*often*”, 5 = “*all of the time*”). Example item are “*I have been feeling useful*” and “*I’ve been feeling relaxed*”. The total score is a mean of the item responses, though the scale was modified by excluding the item *“I have been feeling close to other people”* since it was related to feelings of loneliness (hence, the current scale was 6, not 7, items). Scores could range from 1–5, with higher scores reflecting better positive mental wellbeing, and the scale evidenced good internal reliability (α_T1_ = 0.86; α_T4_ = 0.91).

### Procedure

The Human Research Ethics Committees of The University of Western Australia and the Western Australian Department of Education granted permission to conduct this research. Informed consent was obtained from all individual participants, and their parents/guardians, included in the study. All participants were provided with a unique identification code, which allowed them to log on to an electronic survey at each administration. This unique code also ensured that all information provided was confidential and that data could be linked across waves via these codes for the purposes of data analysis. Data were collected on six occasions, four of which are relevant to the current study: T1 (November/December, 2013: Loneliness, Symptoms of Depression, and Positive Mental Wellbeing all assessed), T2 (August/September, 2014; Loneliness assessed), T3 (March/April, 2015; Loneliness assessed), and T4 (August/September, 2015: Loneliness, Symptoms of Depression, and Positive Mental Wellbeing all assessed). In addition to the aforementioned measures, young people’s screen use was also assessed and those data are reported elsewhere (see Houghton et al., 2015; Rosenberg et al., 2018).

One teacher in each school was responsible for liaising with the research team and for administering the survey. These teachers were provided with written instructions to ensure standardisation of administration. The electronic survey remained open for ~4 weeks across each of the test administrations. Close monitoring of the survey administrations resulted in attrition rates being maintained below 6% per data collection period.

### Statistical Analyses

MPlus Version 8.7 was used to perform a latent trajectory analysis for both friendship-related loneliness and isolation-related loneliness measures over four time points. As only four time points were available, trajectories were fitted as linear across the four waves. Latent trajectory analysis identifies distinct subgroups within the cohort who have similar trajectories of friendship-related loneliness or isolation-related loneliness across waves. The appropriate number of classes for both loneliness measures was determined by starting with an unconditional model with no latent trajectory classes, and conducting subsequent analyses adding one trajectory class at a time and comparing fit. Intercept and slope were permitted to vary across trajectory classes. The most appropriate number of classes was chosen using a combination of (i) smaller Bayesian information criterion (BIC), (ii) higher entropy (>0.8) (Asparouhov & Muthén (2014)). MPlus implements Full Information Maximum Likelihood (FIML) to address missing data.

To investigate the relationship between latent trajectories of loneliness and measures of wellbeing, generalised linear modelling was undertaken using the GENMOD procedure in SAS Version 9.4. Four separate models were fitted, two with CDI depression score (without loneliness item) at Wave 4 as outcome variable, and two with WEMWBS score (without loneliness item) at Wave 4 as outcome variable. In each case the model adjusted for age, gender, and CDI or WEMWBS score at Wave 1. Because of the high degree of correlation between friendship related loneliness and isolation loneliness, separate models were fitted with friendship related loneliness trajectory class or isolation loneliness trajectory class as independent variable.

Sensitivity analyses were also conducted by restricting the aforementioned analyses to those adolescents who only participated in all four waves of data collection. This involved a smaller sample of 1212 young people.

## Results

### Trajectories of Loneliness

**Fig. 1:**
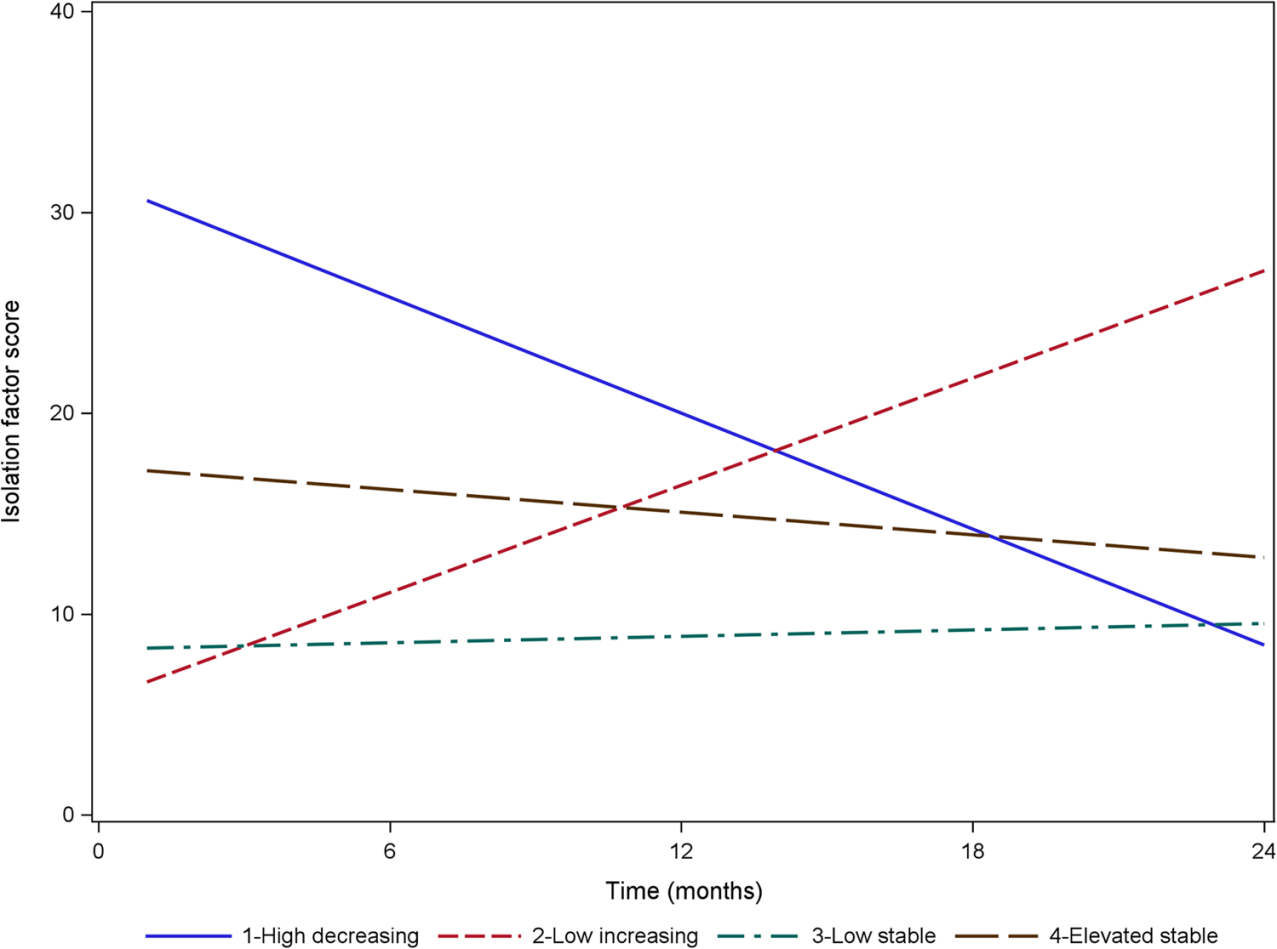
Isolation loneliness trajectories

**Fig. 2:**
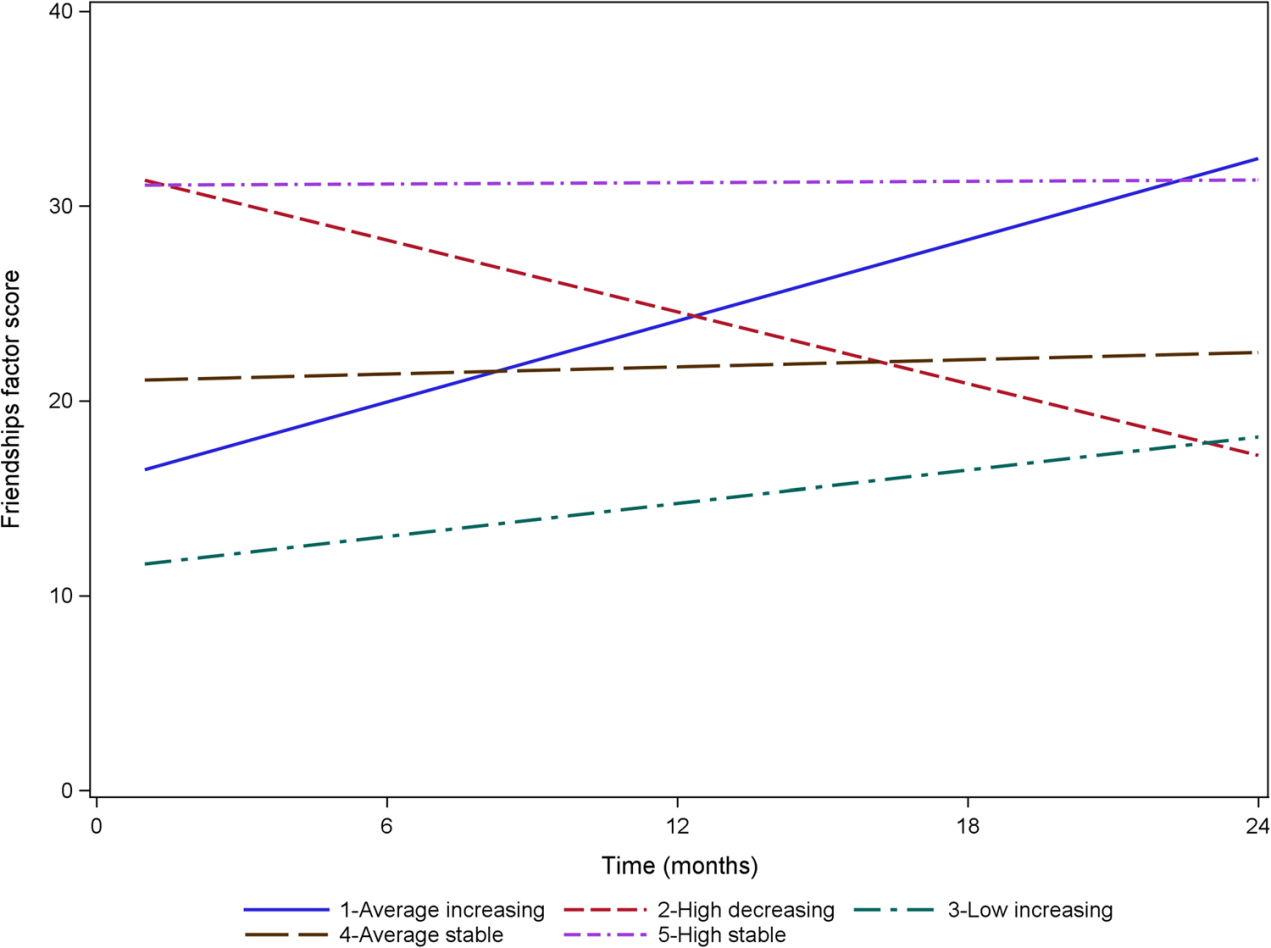
Friendship loneliness trajectories

For Isolation Loneliness, four trajectories were identified (see Table 2, and Fig. 1): Low Stable (74.7%), Elevated Stable (17.4%), Low Increasing (5.1%), and High Decreasing (2.8%). For Friendship Loneliness, there were five groups: High Stable (54.5%), Average Stable (25.9%), High Decreasing (12.5%), Average Increasing (3.8%), and Low Increasing (3.3%) (see Table 2, and Fig. 2). When class membership relating to both forms of loneliness is examined (see Table 3), the most common category combinations are High Stable Friendship + Low Stable Isolation (48.2% of all participants), Average Stable Friendship + Low Stable Isolation (14.7%), Average Stable Friendship + Elevated Stable Isolation (10.0%), and High Decreasing Friendship + Low Stable Isolation (8.7%). All other category combinations included less than 3% of the total sample. Sensitivity analyses confirmed the same optimal number of classes among young people who were present at all four data points and that these were comparable (see Online Supplement 1).

**Table 2:** Entropy and Bayesian Information Criterion (BIC) relating to number of loneliness trajectories for both Friendship Loneliness and Isolation Loneliness subscales

Number of trajectories	Friendship Loneliness				Isolation Loneliness			
	Entropy	BIC	Change in BIC		Entropy	BIC	Change in BIC	
			*p*1	*p*2			*p*1	*p*2
2	0.608	39538	0.0000	0.0000	0.823	37226	0.0000	0.0000
3	0.637	39447	0.0000	0.0000	0.877	36950	0.0028	0.0035
4	0.640	39398	0.0045	0.0054	0.883	36654	0.0000	0.0000
5	0.689	39371	0.0028	0.0035	0.813	36562	0.0903	0.0917
6	0.668	39359	0.2462	0.2628	-	-	-	-

Change in BIC was assessed using the Vuong-Lo_Mendell-Rubin Likelihood Ratio Test (p1 above) and the Lo-Mendell-Rubin Adjusted Likelihood Ratio Test (p2 above)

**Table 3:** Number of participants in each loneliness trajectories for both Friendship Loneliness and Isolation Loneliness subscales

	Isolation Loneliness				Row total (Column %)
	High Decreasing	Low Increasing	Low Stable	Elevated Stable	
Friendship Loneliness					
Average Increasing	4	5	36	26	71 (3.8%)
High Decreasing	14	22	164	35	235 (12.5%)
Low Increasing	7	7	21	27	62 (3.3%)
Average Stable	9	14	276	189	488 (25.9%)
High Stable	19	48	908	51	1026 (54.5%)
Column total (Row %)	53 (2.8%)	96 (5.1%)	1405 (74.7%)	328 (17.4%)	

#### Descriptive statistics

In Table 4, descriptive statistics for the measures of positive mental wellbeing and depressive symptomatology at the start and end of the study are shown by trajectory class. For symptoms of depression, the largest changes in mean scores were evident among the Average Increasing and High Decreasing Friendship related loneliness trajectory groups, though these changes occurred within the context of substantial variation in scores at each time point. For the Isolation loneliness trajectory groups, the High Decreasing and Low Increasing groups showed most change and, even in the context of high variation in scores, the Low Increasing group increased by a full standard deviation.

**Table 4:** Descriptive statistics for positive mental wellbeing and depressive symptomatology at start and end of the study, by trajectory class

Loneliness Trajectory Group	Depression				Positive Mental Wellbeing			
	T1		T4		T1		T4	
	Mean	S.D.	Mean	S.D.	Mean	S.D.	Mean	S.D.
Friendship Subscale								
Average Increasing	5.27	4.43	3.81	3.99	2.21	0.71	2.82	0.48
High Decreasing	3.74	3.53	6.50	5.16	2.63	0.55	2.15	0.72
Low Increasing	8.80	5.04	9.03	4.54	1.82	0.80	1.95	0.61
Average Stable	5.61	3.60	5.60	4.30	2.28	0.46	2.35	0.53
High Stable	2.69	2.78	2.83	3.12	2.74	0.46	2.83	0.48
Isolation Subscale								
High Decreasing	7.43	6.53	5.22	5.25	2.59	0.78	2.46	0.76
Low Increasing	4.40	4.40	8.86	6.75	2.54	0.62	2.47	0.91
Elevated Stable	6.25	3.77	6.30	4.43	2.22	0.53	2.30	0.61
Low Stable	3.11	2.97	3.40	3.43	2.64	0.52	2.67	0.56

For positive wellbeing, the same two Friendship related loneliness trajectory groups again showed the largest changes and these changes were of almost a full standard deviation. Finally, for Isolation Loneliness, there were only relatively small changes observed.

As before, sensitivity analyses confirmed that the classes had very similar characteristics to the previously derived classes (see Online Supplement 2).

### Associations with Depressive Symptomatology at T4 Controlling for T1 Depressive Symptomatology

The Low Stable Isolation Loneliness group was used as the reference group when estimating possible effects relating to Isolation Loneliness, as this was the most frequently occurring group (74.7% of youth). Similarly, when estimating effects for Friendship Isolation, the High Stable Friendship group was used as the reference group (54.5% of youth). Across both analyses, age did not predict depressive symptomatology, but girls reported significantly more symptoms of depression than boys (see Table 5 for results).

**Table 5:** Results of generalised linear modelling analyses investigating the relationship between latent trajectories of loneliness and measures of wellbeing^a^

Loneliness Trajectory Group /Covariate	Depression		Positive Mental Wellbeing	
	Estimate^b^	Sig	Estimate^b^	Sig
Friendship Subscale				
Average Increasing	−0.16 (−1.25, 0.93)	0.771	0.13 (−0.02, 0.28)	0.095
High Decreasing	3.35 (2.69, 4.01)	<0.001	−0.64 (−0.73, −0.55)	<0.001
Low Increasing	4.46 (2.97, 5.95)	<0.001	−0.71 (−0.91, −0.52)	<0.001
Average Stable	1.84 (1.26, 2.43)	<0.001	−0.37 (−0.45, −0.29)	<0.001
High Stable	*Reference category*			
Gender				
Males	−0.86 (−1.31, −0.41)	<0.001	0.15 (0.09, 0.21)	<0.001
Females	*Reference category*			
Age (years) at wave 1	0.13 (−0.01, 0.27)	0.078	−0.02 (−0.04, 0.01)	0.061
Depression at wave 1	0.33 (0.26, 0.40)	<0.001		
Positive Mental Wellbeing at Wave 1			0.28 (0.22, 0.34)	<0.001
Isolation Subscale				
High Decreasing	1.68 (0.29, 3.07)	0.018	−0.24 (−0.50, −0.00)	0.012
Low Increasing	5.33 (4.32, 6.35)	<0.001	−0.19 (−0.34, −0.04)	0.015
Elevated Stable	1.48 (0.85, 2.10)	<0.001	−0.18 (−0.27, −0.08)	<0.001
Low Stable	*Reference category*			
Gender				
Males	−0.84 (−1.29, −0.38)	<0.001	0.14 (0.07, 0.21)	0.035
Females	*Reference category*			
Age (years) at wave 1	0.09 (−0.04, 0.24)	0.183	−0.03 (−0.05, −0.01)	0.015
Depression at wave 1	0.37 (0.30, 0.44)	<0.001		
Positive Mental Wellbeing at Wave 1			0.35 (0.28, 0.41)	<0.001

^a^Age, Gender, and Baseline score on the relevant dependent variable were all controlled for as covariates

^b^Estimates are unstandardised and include confidence intervals

#### Isolation loneliness

Compared to the reference group, all other groups reported significantly higher levels of depressive symptomatology. The biggest effect was observed when comparing the Low Increasing trajectory group (i.e. Isolation Loneliness worsening) where the model estimates a difference between these groups of more than 5 points, a large effect given that the scale only ranged from 0–20. As shown in Fig. 1, this group finished with the highest loneliness scores. The other two groups (High Decreasing and Elevated Stable) were approximately 1.5 points higher than the Low Stable group.

#### Friendship related loneliness

The reference group reported significantly fewer symptoms of depression that the High Decreasing Friendship Loneliness group, Low Increasing Friendship Loneliness group, and Average Stable Friendship Loneliness group. However, the reference group did not differ from the Average Increasing Friendship Loneliness group. The biggest effect was evident when comparing the Low Increasing Friendship Loneliness group to the reference group, with an estimated difference on final depressive symptomatology of 4.46 points. The High Decreasing Friendship Loneliness group was also significantly higher than the High Stable group by 3.35 points.

### Associations with Positive Mental Wellbeing at T4 Controlling for T1 Positive Mental Wellbeing

The same reference groups were again employed in analyses relating to positive wellbeing (see section 3.2). Across both analyses, boys reported significantly higher levels of positive wellbeing than girls, though only in the Isolation Loneliness model did age negatively predict positive wellbeing (see Table 3 for results).

#### Isolation loneliness

All three comparison groups reported significantly lower levels of positive mental wellbeing at the end of the study as compared to the reference group. Estimated reductions ranged from 0.18 to 0.24 (on a scale of 1–5).

#### Friendship related loneliness

The High Decreasing, Low Increasing, and Average Stable Friendship Loneliness groups all reported significantly lower positive mental wellbeing than the reference group. These reductions ranged from 0.37 to 0.71. However, the Average Increasing group did not differ from the reference group.

Sensitivity analyses again provided results with aligned with the main results reported above (see Online Supplement 2). Only one parameter was more than trivially different, and this was still non-significantly different. Low Increasing friendship subscale as a predictor or depression, co-efficient changed from 4.46 (2.97–5.95) to 2.52 (1.03–4.00).

## Discussion

There is a need to identify the outcomes of changes in loneliness during adolescence, and to consider this within a multidimensional framework of loneliness. When experienced in adolescence, loneliness can impose long-term distress and significant adverse outcomes in later years, regardless of whether it recurs or persists over time (Matthews et al., 2023). Therefore, the importance of interventions to break the cycle of loneliness during the early years is critical. To achieve this, the present study identified trajectories of change in relation to two dimensions of loneliness (Isolation loneliness and Friendship loneliness) and considered the effects of these on both positive mental wellbeing and symptoms of depression. Expectations relating to the number of trajectories (four or five), and the form that trajectories would take, were largely confirmed. Identification of different numbers of trajectory groups for each of the two different forms of loneliness is consistent with a multidimensional conceptualisation of loneliness. Additionally, youth in different trajectory groups evidenced clear differences in their mental health outcomes, supporting the argument that it is important to consider change and development in loneliness.

It was hypothesised that there would exist four or five trajectories of loneliness. Four trajectories were found for Isolation loneliness and five trajectories for Friendship related loneliness. The largest trajectory group in both cases, and as expected, is a consistently unproblematic one: 75% of youth were in the Low Stable Isolation Loneliness trajectory groups and 55% were in the High Stable Friendship Loneliness trajectory (with a further 26% reporting Average Stable Friendship Loneliness). However, contrary to expectation (Ladd & Ettekal, 2013; Qualter et al., 2013; Schinka et al., 2013; Vanhalst et al., 2013, 2015, 2018), there was not a chronically high trajectory for Isolation loneliness, though in line with expectations there was a group of adolescents who reported consistently low levels of Friendship related loneliness. Given the relationships between trajectory membership and adjustment, discussed below, these prevalence rates are very encouraging because they indicate that most youth were not experiencing problematic levels of loneliness and were not therefore at risk for the problematic outcomes associated with such membership.

Since this is the first report of trajectories for two separate loneliness factors, the ways in which these may co-occur was documented. Four combinations were most prevalent: Low Stable Isolation Loneliness with either High Stable Friendship Loneliness (48%), High Decreasing Friendship Loneliness (9%), or Average Stable Friendship Loneliness (15%), and Elevated Stable Isolation Loneliness with Average Stable Friendship Loneliness (10%). All other trajectory group combinations occur at a prevalence of under 3%, though there were at least some young people in all 20 possible combination of trajectories. These combinations speak to the need for theory to consider the ways in which dimensions of loneliness may interact to produce outcomes. Of particular interest here is the possible disparity between desired and experienced levels of loneliness (Cacioppo et al., 2015b; Qualter et al., 2013; Rook, 1984) as the existence of these combinations suggests that youth may hold different beliefs about such a mismatch across different factors of loneliness. Alternatively, it may be that one or other dimension is associated with shorter, or fewer, fluctuations over time, and experience sampling assessments (e.g., van Roekel et al., 2014) offer appropriate ways to assess such a proposition in future. However, it is encouraging to see that the most prevalent group is that which combines both of the least problematic trajectories of the two forms of loneliness.

These results speak to the multidimensionality of loneliness (Goossens et al., 2009; Houghton et al., 2014; Majorano et al., 2015). Different numbers of trajectory classes for each type of loneliness is evidence in support of a multidimensional conceptualisation. In addition, youth did not always report “equivalent” trajectories on each measure of loneliness as might be expected if the two scales were simply mirror-images of one another. For example, only 23% of young people in the Low Increasing Isolation Loneliness trajectory were also in the High Decreasing Friendship Loneliness trajectory. Similarly, only 13% of young people in the High Decreasing Isolation Loneliness trajectory were also in the Low-Increasing Friendship Loneliness trajectory. These results strengthen the case for considering Friendship loneliness and Isolation loneliness as distinctive dimensions, reflecting the importance of considering both the quality of friendships and the degree to which youth feel isolated from others.

The benefits of having stable, low trajectories of loneliness with respect to symptoms of depression have been reported in previous research (e.g., Harris et al., 2013; Qualter et al., 2013; Schinka et al., 2013; Vanhalst et al., 2013). This was echoed on the current findings, with the Low Stable Isolation Loneliness trajectory group reporting fewer symptoms of depression than all other youth in this study. The difference was particularly notable concerning other youth in the Low Increasing Isolation loneliness trajectory group, whose levels of depressive symptomatology increased by more than a standard deviation from the start to the end of the study. The reasons underpinning why young people experiencing *increasing* levels of loneliness across adolescence are at particular risk of experiencing symptoms of depression is unknown, but the disconnection between desired and perceived relationships which is the core of loneliness (e.g. Cacioppo et al., 2015b) may offer some insight. Specifically, an increasing awareness of how far one’s desires are from what one actually has, especially in the context of having had it previously, may differentiate this group from those who experience decreasing, or consistently high, levels of Isolation Loneliness. Such a pattern of results could provide support for a scar hypothesis (Rohde et al., 1990) pertaining to loneliness. Future research should seek to interrogate this possibility in more detail.

Novel findings reported here also indicated that specific trajectories of loneliness during adolescence can negatively impact positive mental wellbeing even after controlling for earlier levels of that construct. Outcomes for positive mental wellbeing were very similar to those reported above for symptoms of depression, although the risk associated with the Low Increasing Isolation loneliness trajectory group for symptoms of depression was not apparent for positive mental wellbeing. This is the first research to establish a link between trajectories of loneliness during adolescence and low levels of positive mental health and extends results reported in correlational studies (Houghton et al., 2016; Lyyra et al., 2021). While the present results contrast with Houghton et al.’s (2022), the unique context of the COVID-19 pandemic that took place during that study may have had an impact on their results. Thus, interventions that can successfully tackle loneliness (Eccles & Qualter (2021)) may usefully be used alongside, or integrated into, existing interventions to enhance positive mental wellbeing for young people (see Cilar et al., 2020).

The results reported here may not generalise to adolescents outside of Western Australia. In addition, a reliance on self-reports may mean the results are subject to limitations relating to mono-method approaches such as shared-method variance. However, accurate and reliable reports of subjective dispositions such as those which were the subject of the current study can be difficult to obtain from third parties such as teachers or parents (see Frick et al., 2009). The number of trajectories for each of the two forms of loneliness that were investigated here may be sample-specific, though one of the strengths of the study was the recruitment of a large demographically representative sample, a longitudinal design with low levels of attrition, and the use of state-of-the-art statistical techniques. A final limitation relates to the collection of both loneliness and outcome data at the same final time point, which may restrict causal interpretations of these data.

## Conclusion

Loneliness can be best described as multidimensional during adolescence, yet there is little understanding of the ways in which different dimensions of loneliness develop during this period. The results of this study imply that change and stability in these dimensions are differentially associated with fluctuations in symptoms of depression and positive mental health across a 2-year period. Young people who experienced low levels of Isolation Loneliness that subsequently increased, and those for whom their quality of friendships were consistently low (despite increases), appear to be at particular risk for poor outcomes. Taken together, it appears that being cognisant of the ways in which loneliness is experienced over time is an important additional consideration when seeking to influence young people’s mental health.

### Supplementary Information

Online Supplement 1

Online Supplement 2
