# Supplementary material for: Trajectories of Loneliness During Adolescence Predict Subsequent Symptoms of Depression and Positive Wellbeing
Source: J Youth Adolesc. 2023 Dec 21;53(5):1078–90. doi: 10.1007/s10964-023-01925-0 (PMC10980621; doi:10.1007/s10964-023-01925-0)

Trajectories of Loneliness During Adolescence Predict Subsequent Symptoms of Depression and Positive Wellbeing

**Online Supplement 1**

Entropy and Bayesian Information Criterion (BCI) relating to number of loneliness trajectories for both Friendship Loneliness and Isolation Loneliness subscales^*^.

| Number of trajectories | Friendship Loneliness | | | Isolation Loneliness | | |
| --- | --- | --- | --- | --- | --- | --- |
|  | Entropy | Change in BIC | | Entropy | Change in BIC | |
|  |  | p1 | p2 |  | p1 | p2 |
| 2 | 0.674 | 0.0000 | 0.0000 | 0.928 | 0.0000 | 0.0000 |
| 3 | 0.680 | 0.0000 | 0.0000 | 0.930 | 0.0006 | 0.0008 |
| 4 | 0.686 | 0.0294 | 0.0337 | 0.917 | 0.0068 | 0.0080 |
| 5 | 0.748 | 0.0026 | 0.0032 | 0.880 | 0.0957 | 0.1023 |
| 6 | 0.716 | 0.104 | 0.126 | - | - | - |

Note: Change in BIC was assessed using the Vuong-Lo_Mendell-Rubin Likelihood Ratio Test (p1 above) and the Lo-Mendell-Rubin Adjusted Likelihood Ratio Test (p2 above).

* Based on n=1,212 adolescents participating in all four waves

Confirmed optimal number of classes:

Friendship loneliness: 5

Isolation loneliness: 4

**Figure 1: Isolation loneliness trajectories**


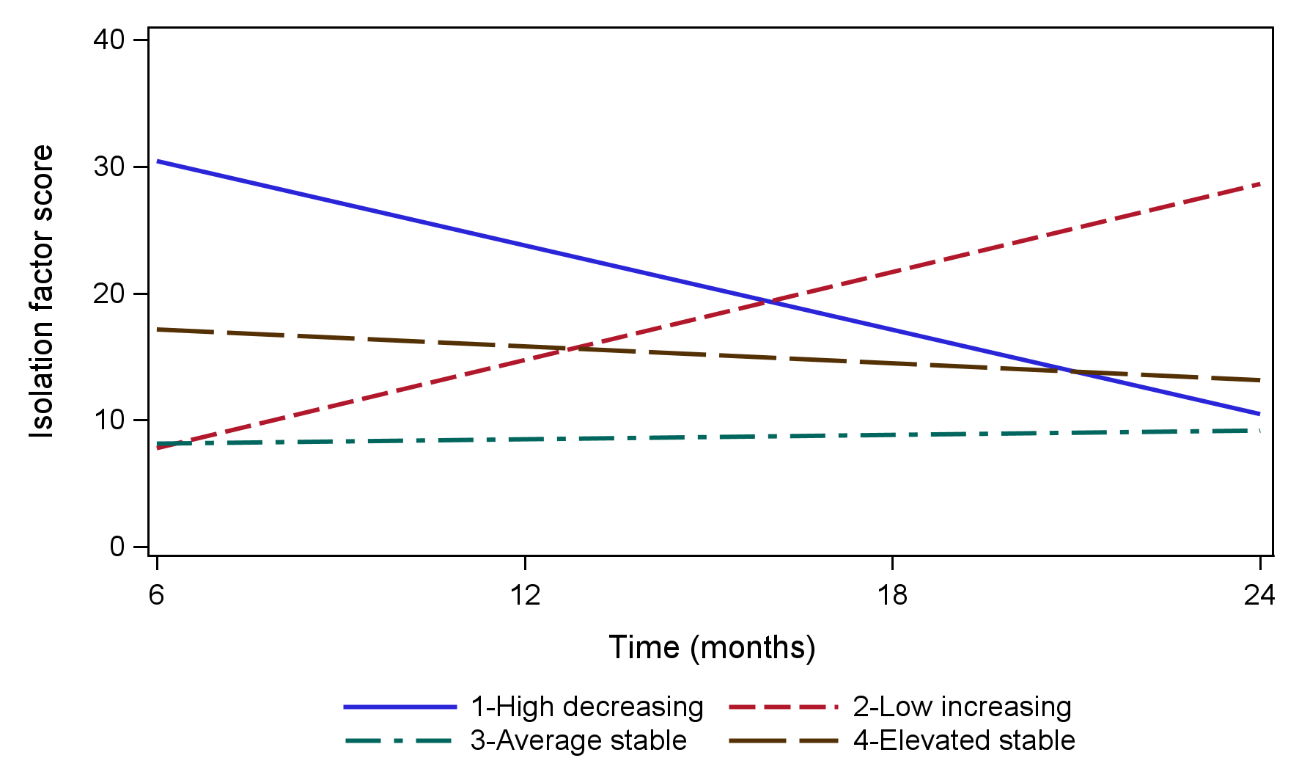


**Figure 2: Friendship loneliness trajectories**


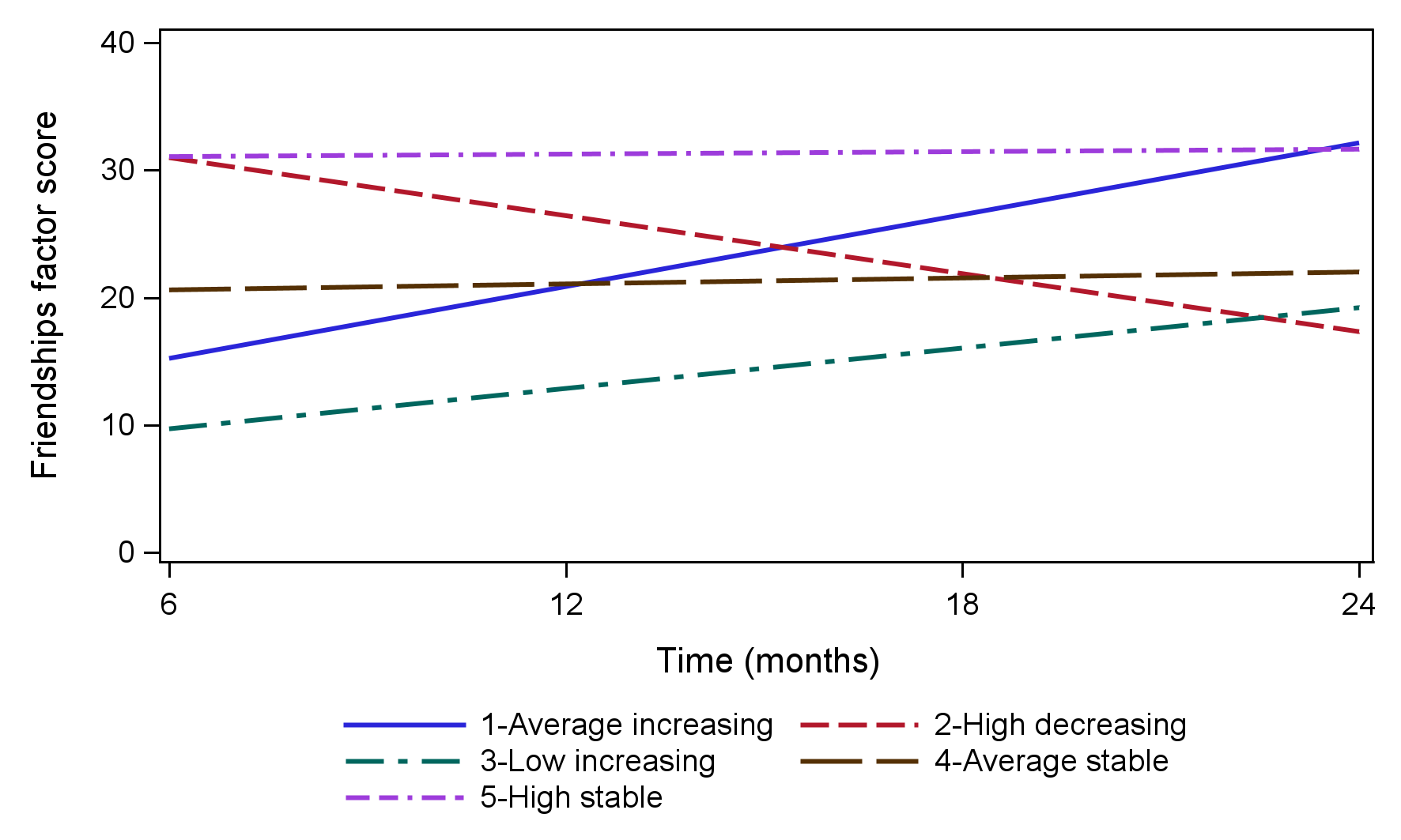

Supplement: Supplementary file 1 — Online Supplement 1 [file 10964_2023_1925_MOESM1_ESM.docx]
