# Supplementary material for: Trajectories of Loneliness During Adolescence Predict Subsequent Symptoms of Depression and Positive Wellbeing
Source: J Youth Adolesc. 2023 Dec 21;53(5):1078–90. doi: 10.1007/s10964-023-01925-0 (PMC10980621; doi:10.1007/s10964-023-01925-0)
Supplement: Supplementary file 2 — Online Supplement 2 [file 10964_2023_1925_MOESM2_ESM.docx]

Trajectories of Loneliness During Adolescence Predict Subsequent Symptoms of Depression and Positive Wellbeing

**Online Supplement 2**

**Table 1**

Number of participants in each loneliness trajectory for both Friendship Loneliness and Isolation Loneliness subscales.

|  | Isolation Loneliness | | | | Row total  (Column %) |
| --- | --- | --- | --- | --- | --- |
|  | High Decreasing | Low Increasing | Low  Stable | Elevated Stable |  |
| Friendship Loneliness |  |  |  |  |  |
| Average Increasing | 2 | 3 | 25 | 23 | 53 (4.4%) |
| High Decreasing | 12 | 18 | 133 | 19 | 182 (15.0%) |
| Low Increasing | 4 | 3 | 9 | 16 | 32 (2.6%) |
| Average Stable | 5 | 7 | 165 | 112 | 289 (23.8%) |
| High Stable | 14 | 26 | 576 | 40 | 656 (54.1%) |
| Column total | 37 | 57 | 908 | 210 |  |
| (Row %) | (3.0%) | (4.7%) | (74.9%) | (17.4%) |  |

**Table 2**

Descriptive statistics for positive mental wellbeing and depressive symptomatology at start and end of the study, by trajectory class.

| Loneliness Trajectory Group | Depression | | | | Positive Mental Wellbeing | | | |
| --- | --- | --- | --- | --- | --- | --- | --- | --- |
|  | T1 | | T2 | | T1 | | T2 | |
|  | Mean | S.D. | Mean | S.D. | Mean | S.D. | Mean | S.D. |
| Friendship Subscale |  |  |  |  |  |  |  |  |
| Average Increasing | 5.24 | 4.12 | 3.55 | 4.04 | 2.26 | 0.69 | 2.88 | 0.47 |
| High Decreasing | 3.67 | 3.58 | 6.38 | 5.00 | 2.63 | 0.55 | 2.15 | 0.73 |
| Low Increasing | 8.76 | 5.88 | 7.41 | 4.07 | 2.01 | 0.83 | 2.12 | 0.53 |
| Average Stable | 5.35 | 3.49 | 5.45 | 4.30 | 2.30 | 0.44 | 2.35 | 0.53 |
| High Stable | 2.59 | 2.71 | 2.72 | 3.15 | 2.74 | 0.46 | 2.86 | 0.48 |
| Isolation Subscale |  |  |  |  |  |  |  |  |
| High Decreasing | 6.97 | 6.50 | 5.67 | 5.44 | 2.60 | 0.82 | 2.42 | 0.78 |
| Low Increasing | 4.42 | 4.64 | 9.47 | 7.44 | 2.54 | 0.63 | 2.43 | 0.97 |
| Elevated Stable | 6.00 | 3.72 | 5.97 | 4.25 | 2.24 | 0.57 | 2.35 | 0.62 |
| Low Stable | 2.97 | 2.88 | 3.20 | 3.22 | 2.66 | 0.49 | 2.70 | 0.56 |

**Table 3**

Results of generalised linear modelling analyses investigating the relationship between latent trajectories of loneliness and measures of wellbeing^1^.

| Loneliness  Trajectory Group | Depression | | Positive Mental Wellbeing | |
| --- | --- | --- | --- | --- |
|  | Estimate^2^ | Sig | Estimate^2^ | Sig |
| Friendship subscale | | | | |
| Average Increasing | -0.14(-0.24, 0.96) | .798 | 0.18 (0.03, 0.33) | .017 |
| High Decreasing | 3.26 (2.59, 3.93) | <.001 | -0.62 (-0.70, -0.53) | <.001 |
| Low Increasing | 2.52 (1.03, 4.00) | <.001 | -0.59 (-0.79, -0.39) | <.001 |
| Average Stable | 1.68 (1.08, 2.28) | <.001 | -0.35 (-0.43, -0.27) | <.001 |
| High Stable | *Reference category* |  |  |  |
| Isolation subscale | | | | |
| High Decreasing | 1.67 (0.31, 3.03) | .016 | -0.29 (-0.48, -0.10) | .003 |
| Low Increasing | 5.95 (4.94, 6.97) | <.001 | -0.23 (-0.39, -0.08) | .003 |
| Elevated Stable | 1.37 (0.74, 2.00) | <.001 | -0.15 (-0.24, -0.06) | <.001 |
| Low Stable | *Reference category* |  |  |  |

^1^Age, Gender, and Baseline score on the relevant dependent variable were all controlled for as covariates.

^2^ Estimates are unstandardized and include confidence intervals.
